# Effect of Mn, Fe and Co on the compression strength and ductility of in situ nano-sized TiB_2_/TiAl composites

**DOI:** 10.1186/s40064-015-1575-5

**Published:** 2015-12-18

**Authors:** Shili Shu, Cunzhu Tong, Feng Qiu, Qichuan Jiang

**Affiliations:** Key Laboratory of Automobile Materials, Ministry of Education, and Department of Materials Science and Engineering, Jilin University, No. 5988 Renmin Street, Changchun, 130025 People’s Republic of China; State Key Laboratory of Luminescence and Applications, Changchun Institute of Optics, Fine Mechanics and Physics, Chinese Academy of Sciences, Changchun, 130012 People’s Republic of China; Department of Mechanical Engineering, Oakland University, Rochester, MI 48309 USA

**Keywords:** TiAl, Composite, Alloying element, Compression properties

## Abstract

The element of Fe can enhance the strength of TiB_2_/TiAl composite, but it is detrimental to the ductility of the composite due to the existence of large bulk TiB_2_ phase at grain boundary. The element of Mn is beneficial to the ductility of TiB_2_/TiAl composite, of which the fracture strain increases from 15.9 to 17.9 % with the addition of 2 at.% Mn. The element of Co can improve the strength and ductility of TiB_2_/TiAl composite simultaneously. With the addition of 2 at.% Co, the ultimate compression strength of TiB_2_/TiAl composite increases from 1829 to 1906 MPa and the fracture strain increases from 15.9 to 17.2 %.

## Background

In recent 20 years, TiAl alloys have received considerable interest as potential high-temperature structural materials for aerospace, automotive and other applications (Tsujimoto et al. 1992; Kim 1994; Wu 2006). However, its practical application has been limited because of its low ductility at room temperature and insufficient strength at elevated temperatures (Ramaseshan et al. 1999; Huang and Hall 1991; Kumagai and Nakamura 1996).

Composite technology is an effective approach to enhance the strength of materials by introducing stiff and hard particle reinforcements. So, some hard particle reinforcements, such as TiB_2_, Al_2_O_3_, Ti_5_Si_3_ and Ti_2_AlC have been introduced into TiAl alloy for enhancing its strength (Lee and Lee 1999; Hirose et al. 1997; Bohn et al. 2001; Yeh and Li 2008; VanMeter et al. 1996; Yang et al. 2010). VanMeter et al. (1996) fabricated the 40 and 50 vol. % TiB_2_/TiAl composites by the method of powder metallurgy techniques. The compression strength of the composites was reported in the range of 2484–2866 MPa, while the fracture strain was reported in the range of 0.4–1.7 %. Yang et al. (2010) fabricated theTi_2_AlC/TiAl composites by the method of spark plasma sintering (SPS) through a mixture powders with the composition of Ti–50Al (at.%) and 10 vol % carbon nanotubes. It was found that when the SPS temperature was 950 °C, the compression yield strength of sintered sample reached to 2058 MPa, while the fracture strain was about 0.16 %. These above results indicate that the addition of ceramic particles could enhance the strength of TiAl alloy, while is usually deleterious to ductility. In our previous work (Shu et al. 2012, 2013a, b), among the ceramic particles of Ti_2_AlC, TiB_2_ and Ti_5_Si_3_, the TiB_2_ particle possesses the best strengthening effect to TiAl alloy. With the addition of 4 vol. % TiB_2_ particles, the compression strength of TiAl alloy increases from 1415 to 1829 MPa. But the strength enhancement of composite is at the cost of ductility, the fracture strain of TiAl alloy decreases from 17.3 to 15.9 %.

So, base on composite technology, it is necessary to find another method to overcome the ductility deterioration of TiB_2_/TiAl composite, or even to improve its ductility to get it with high strength and good ductility. Element alloying, which is achieved by introducing substitutional atoms into materials, has been proved can improve the ductility of TiAl alloy (Duan et al. 2010; Liu and Nash 2011; Kawabata et al. 1998; Music and Schneider 2006). So, we believe that if the alloying elements, which are beneficial to the ductility of TiAl alloy, are added to TiB_2_/TiAl composite, the composite is hopeful to possess high strength and good ductility simultaneously. According to our previous work (Shu et al. 2013a, b, 2014), it has been confirmed by the theory calculation and experimental study that the alloying elements of Mn, Fe and Co could effectively improve the ductility of TiAl alloy. While, the effect of alloying elements on TiAl matrix composite would be more complex than that in TiAl alloy and whether they are beneficial to the ductility of TiB_2_/TiAl composite has never been investigated until now.

In present study, we try to obtain the TiB_2_/TiAl composite with high strength and good ductility simultaneously through the combined method of composite technology and element alloying, and the effect of Mn, Fe and Co on the compression properties of TiB_2_/TiAl composite has been investigated.

## Experimental

The starting materials were made from commercial powders of Al (99 % purity, ~47 μm), Ti (99.5 % purity, ~25 μm), Mn (99.5 % purity, ~47 μm), Fe (98.5 % purity, ~47 μm), Co (99.5 % purity, ~47 μm) and B (98 % purity, ~3 μm). Elemental powder blends corresponding to 4 vol. % TiB_2_/TiAl with the addition of 2 at.% Mn, 2 at.% Fe and 2 at.% Co, respectively, were mixed sufficiently by ball milling for 8 h at a low speed (~35 rpm) in a conventional planetary ball-miller. Both the pot and the balls were made of stainless steel and the mass ratio of ball to powders was 20:1–25:1. Then the mixed powders were cold pressed into cylindrical performs using a stainless steel die. The powder compact with 28 mm in diameter and approximately 36 mm in height was contained in a graphite mold, which was put into a self-made vacuum thermal explosion furnace. The heating rate of the furnace was about 30 K/min and the temperature in the vicinity of the center of the compact was measured by Ni–Cr/Ni–Si thermocouples. When the temperature measured by thermocouples suddenly rose rapidly, indicating that the sample should be ignited, the sample was quickly pressed just when it was still hot and soft. The pressure (~50 MPa) was maintained for 10 s and then was cooled down to the ambient temperature at the cooling rate of ~10 K/min.

The phase constituents of the composites were examined by X-ray diffraction (XRD, Moldel D/Max 2500PC Rigaku, Japan) with Cu Kα radiation using a scanning speed of 4°/min. The microstructure was studied using scanning electron microscope (SEM, Evo18, Carl Zeiss, Germany) equipped with an energy dispersive spectrometer (EDS). The morphologies of the ceramic particles were observed using a field emission scanning electron microscope (FESEM, JSM 6700F, JEOL, Tokyo, Japan). All specimens for constituent analysis, microstructure observation and compression tests were taken along the ring at the half radius of the fabricated samples.

The cylindrical specimens with a diameter of 3 mm and a height of 6 mm were used for compression tests, and the loading surface was polished parallel to the other one. The uniaxial compression tests were carried out three times for each sample under a servo-hydraulic materials testing system (MTS, MTS 810, USA) with a strain rate of 1 × 10^−4^ s^−1^. The true stress–strain curves were got from engineering stress–strain curves according to the formularies: $\varepsilon_{\text{t}} = - \ln (1 - \varepsilon_{e} )$ and $\sigma_{t} = \sigma_{e} (1 - \varepsilon_{e} )$, where ε_t_ is the true strain, σ_t_ is the true strength, ε_e_ is the engineering strain and σ_e_ is the engineering strength.

## Results and discussion

### Phase identification and microstructures

**Fig. 1:**
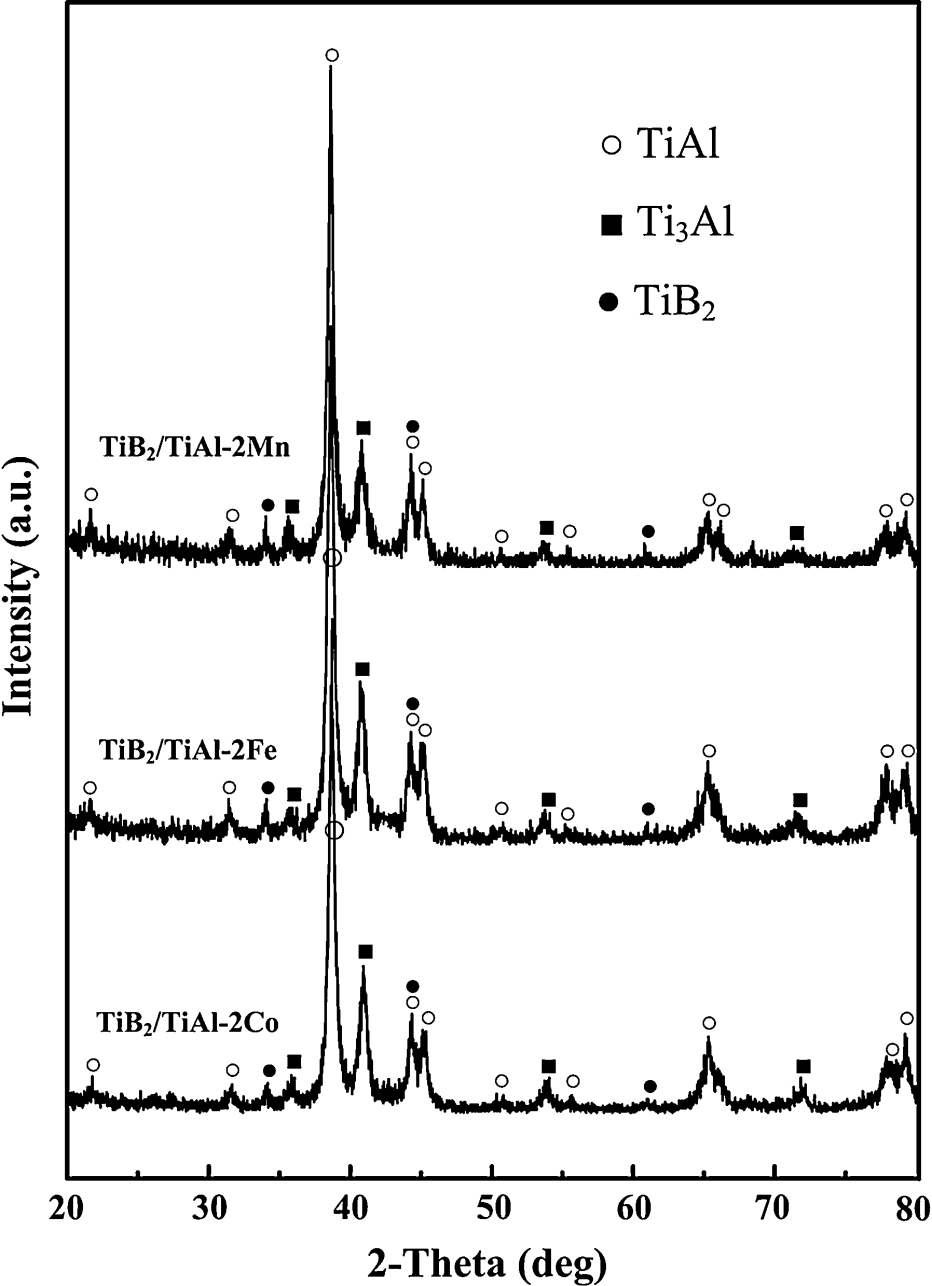
XRD patterns of the TiB_2_/TiAl–2Mn, TiB_2_/TiAl–2Fe and TiB_2_/TiAl–2Co composites, respectively

Figure 1 shows the XRD patterns for the 4 vol. % TiB_2_/TiAl composites with the addition of Mn, Fe and Co elements. The products in these samples are mainly γ-TiAl, α_2_-Ti_3_Al and TiB_2_ phases. The elements of Mn, Fe and Co all have no effect on the phase composition of TiB_2_/TiAl composite. Thus, the existence of these alloying elements is thought to be in the form of solid solution in TiAl matrix. In order to confirm this, the analysis of microstructure and energy–dispersive spectra (EDS) of these samples were conducted.

**Fig. 2:**
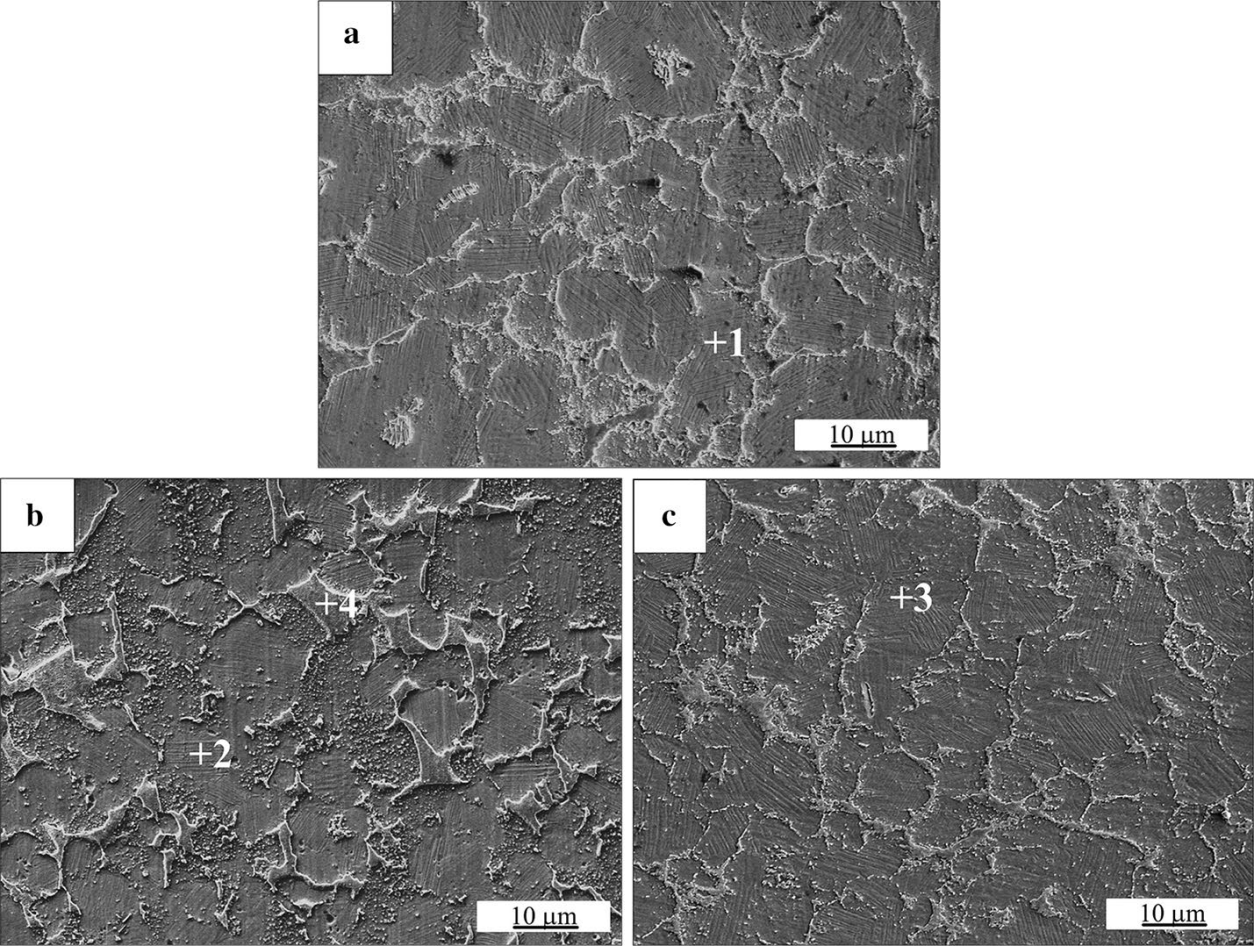
Microstructures of the **a** TiB_2_/TiAl–2Mn, **b** TiB_2_/TiAl–2Fe and **c** TiB_2_/TiAl–2Co composites, respectively

Figure 2a–c show the microstructures of the TiB_2_/TiAl composites with the addition of Mn, Fe and Co elements, respectively. The energy–dispersive spectra (EDS) results of the TiAl matrix detected at points +1, +2 and +3 are listed in Table 1. With the addition of 2 at.% Mn, Fe and Co elements, the actual concentrations of Mn, Fe and Co in TiAl matrix are 1.68, 1.55 and 1.42 at.%, respectively. Thus, it can be confirmed that the elements of Mn, Fe and Co are mainly existed in TiAl matrix in the form of solid solution.

**Table 1:** Results of the energy-dispersive spectra in the TiB_2_/TiAl–2Mn, TiB_2_/TiAl–2Fe and TiB_2_/TiAl–2Co composites

	Ti (at.%)	Al (at.%)	B (at.%)	Mn (at.%)	Fe (at.%)	Co (at.%)
Point 1	74.42	23.90	–	1.68	–	–
Point 2	71.41	27.05	–	–	1.55	–
Point 3	73.70	24.88	–	–	–	1.42
Point 4	44.79	4.68	50.53	–	–	–

It can be seen from Fig. 2a and c that the synthesized TiB_2_ particles in the TiB_2_/TiAl–2Mn and TiB_2_/TiAl–2Co composites all distribute at the grain boundary of the TiAl matrix. As reported in our previous study (Shu et al. 2013a, b), the TiB_2_ particles in the TiB_2_/TiAl composite without any alloying element addition also distributed at the grain boundary of TiAl matrix. So the elements of Mn and Co both have no effect on the distribution of TiB_2_ particles. While, in the TiB_2_/TiAl–2Fe composite, there are large bulk phases existed at the grain boundary of TiAl matrix. In order to confirm the compositions of these bulk phases, the analysis of EDS was conducted on them (pointed as +4). As shown in Table 1, it can be seen that the large bulk phases mainly consist the elements of Ti and B, there is no any trace of the element of Fe. So, the large bulk phases are the compound containing boron and titanium. According to the EDS and XRD results, it can be confirmed that the large bulk phases are also TiB_2_. The addition of Fe element has affected the distribution of TiB_2_ particles.

Moreover, according to the SEM images, the grain sizes of the composites were statistic evaluated by the method of image analysis using the software of Image-Pro Plus. The grain sizes of the TiB_2_/TiAl composites with the addition of the elements of Mn, Fe and Co are all about 10 μm. In our previous study (Shu et al. 2013a, b), the grain size of the 4 vol. % TiB_2_/TiAl composite is about 18 μm. Thus, the elements of Mn, Fe and Co all can refine the grain size of TiB_2_/TiAl composite.

### Compression properties

**Fig. 3:**
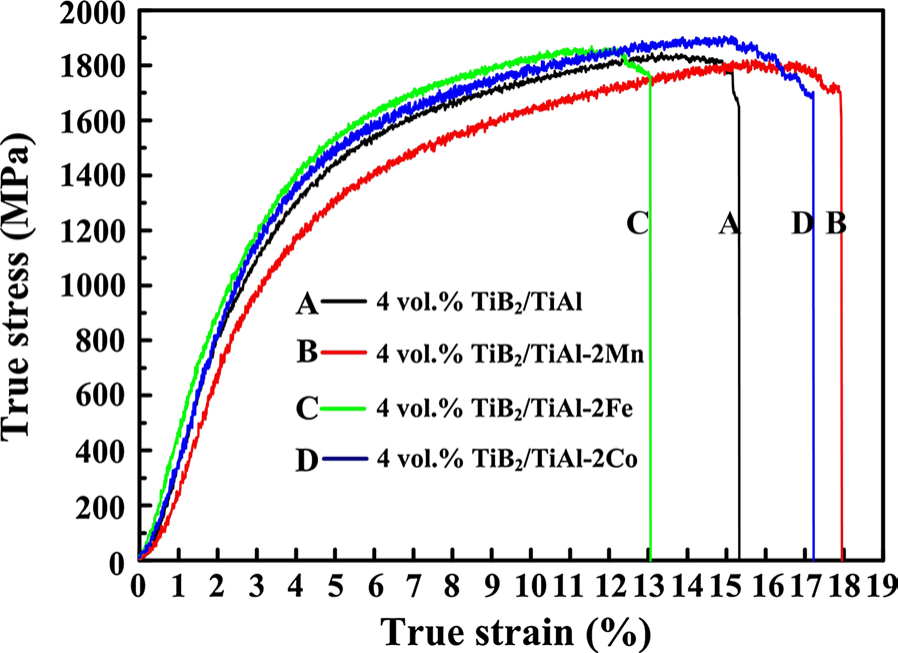
Compression true stress–strain curves of the TiB_2_/TiAl composites with and without element addition

Figure 3 shows the compression true stress–strain curves of the TiB_2_/TiAl composites with and without elements addition. The compression properties of them are summarized in Table 2. It can be seen that the element of Fe could enhance the compression true yield strength ($\sigma_{{\text{true}}}^{\text{y}}$) and the ultimate compression true strength ($\sigma_{true}^{UCS}$) of TiB_2_/TiAl composite, but it is detrimental to the ductility of the composite. With the addition of 2 at.% Fe, the $\sigma_{{\text{true}}}^{\text{y}}$ and $\sigma_{true}^{UCS}$ of the composite increase from 734 and 1829 MPa to 790 and 1878 MPa, respectively, while the fracture strain ($\varepsilon_{{\text{true}}}^{\text{f}}$) decreases from 15.9 to 13.9 %. In our previous study (Shu et al. 2014), the effect of Fe was confirmed to be beneficial to the ductility of TiAl alloy by both theory calculation and experimental study. But for the composite, the distribution of ceramic particles is a very important parameter to its ductility. Thus, the reason for the decrease of the ductility of the TiB_2_/TiAl composite with the addition of Fe is mainly due to the existence of the large bulk TiB_2_ phases at the grain boundary of TiAl matrix.

**Table 2:** Compression properties of the TiB_2_/TiAl–2Mn, TiB_2_/TiAl–2Fe and TiB_2_/TiAl–2Co composites

Sample	$\sigma_{{\text{true}}}^{\text{y}}$ (MPa)	$\sigma_{true}^{UCS}$ (MPa)	$\varepsilon_{{\text{true}}}^{\text{f}}$ (%)
TiAl	465 ± 41	1415 ± 20	17.3 ± 0.03
TiB_2_/TiAl	734 ± 5	1829 ± 17	15.9 ± 0.58
TiB_2_/TiAl–2Mn	785 ± 8	1834 ± 14	17.9 ± 0.21
TiB_2_/TiAl–2Fe	790 ± 11	1878 ± 6	13.9 ± 0.85
TiB_2_/TiAl–2Co	820 ± 3	1906 ± 2	17.2 ± 0.02

While for the elements of Mn and Co, they are both beneficial to the ductility of TiB_2_/TiAl composite. With the addition of 2 at.% Mn and Co, the $\varepsilon_{{\text{true}}}^{\text{f}}$ of the composite increases from 15.9 to 17.9 % and 17.2 %, respectively. Because the elements of Mn and Co both have no effect on the distribution of TiB_2_ particles in the composite, the effect mechanism of Mn and Co on the ductility of composite is the same as in TiAl alloy as reported in our previous work (Shu et al. 2013a, b, 2014). Moreover, with the addition of 2 at.% Co, the $\sigma_{{\text{true}}}^{\text{y}}$ and $\sigma_{true}^{UCS}$ of the composite increase from 734 and 1829 MPa to 820 and 1906 MPa, respectively. The enhancement of the strength with the addition of Co is mainly due to its solid solution strengthening and the grain refinement of TiAl matrix. The element of Co could improve the strength and ductility of TiB_2_/TiAl composite simultaneously. Thus, the TiB_2_/TiAl–2Co composite possesses the highest strength and best ductility, the $\sigma_{{\text{true}}}^{\text{y}}$, $\sigma_{true}^{UCS}$ and $\varepsilon_{{\text{true}}}^{\text{f}}$ of TiB_2_/TiAl–2Co composite are 820, 1906 MPa and 17.2 %, respectively.

**Fig. 4:**
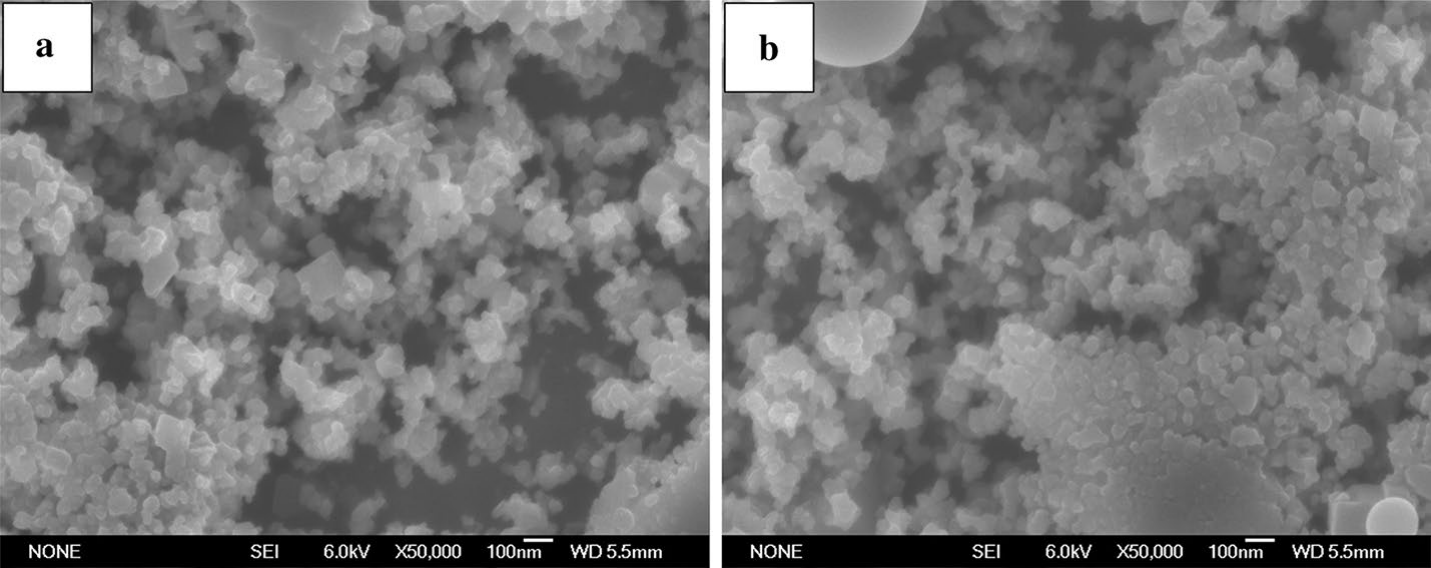
FESEM images of the TiB_2_ particles formed in the **a** TiB_2_/TiAl–2Mn and **b** TiB_2_/TiAl–2Co composites, respectively

The $\sigma_{{\text{true}}}^{\text{y}}$, $\sigma_{true}^{UCS}$ and $\varepsilon_{{\text{true}}}^{\text{f}}$ of the TiAl alloy fabricated in our previous study are 465, 1415 MPa and 17.3 %, respectively (Shu et al. 2013a, b). As compared with the compression properties of the TiAl alloy, it can be seen that with the combined addition of in situ TiB_2_ particles and the elements of Mn or Co could significantly enhance the strength of TiAl alloy with no sacrifice in ductility. The $\sigma_{{\text{true}}}^{\text{y}}$ and $\sigma_{true}^{UCS}$ of the 4 vol. % TiB_2_/TiAl–2Mn composite are 320 and 419 MPa higher than those of TiAl alloy, and the $\sigma_{{\text{true}}}^{\text{y}}$ and $\sigma_{true}^{UCS}$ of the 4 vol. % TiB_2_/TiAl–2Co composite are 355 and 491 MPa higher than those of TiAl alloy. Figure 4a and b show the FESEM images of the TiB_2_ particles formed in TiB_2_/TiAl–2Mn and TiB_2_/TiAl–2Co composites, respectively. It can be seen that the sizes of the TiB_2_ particles formed in these two composites are all in the range of 30–50 nm. It is thought that the solid solution strengthening of Mn or Co, the in situ synthesized nano-TiB_2_ particles and their uniform distribution in the TiB_2_/TiAl–2Mn and TiB_2_/TiAl–2Co composites would be the main reason for the significant strength enhancement without sacrificing ductility. It indicates that the combined method of composite technology and element alloying is an effective way to solve the problem of insufficient strength of TiAl alloy with no sacrifice in ductility.

## Conclusions

The elements of Mn, Fe and Co all mainly exist in TiAl matrix in the form of solid solution, and they all can refine the grain size of TiB_2_/TiAl composite. The elements of Mn and Co both have no effect on the distribution of TiB_2_ particles. While, with the addition of the element of Fe, TiB_2_ particles exist at the grain boundary of TiAl matrix in the form of large bulk. Thus, the element of Fe enhances the strength of TiB_2_/TiAl composite at the cost of ductility. The elements of Mn and Co could both improve the ductility of TiB_2_/TiAl composite. Moreover, the element of Co could also enhance the strength of TiB_2_/TiAl composite. With the addition of 2 at.% Co, the ultimate compression strength of TiB_2_/TiAl composite increases from 1829 to 1906 MPa and the fracture strain increases from 15.9 to 17.2 %. Compared with TiAl alloy, it is also confirmed that the combined method of composite technology and element alloying is an effective way to solve the problem of insufficient strength of TiAl alloy with no sacrifice in ductility.

## References

[CR1] Bohn R, Klassen T, Bormann R (2001). Room temperature mechanical behavior of silicon-doped TiAl alloys with grain sizes in the nano-and submicron-range. Acta Mater.

[CR2] Duan QQ, Luan QD, Liu J, Peng LM (2010). Microstructure and mechanical properties of directionally solidified high-Nb containing Ti-Al alloys. Mater Des.

[CR3] Hirose A, Hasegawa M, Kobayashi KF (1997). Microstructures and mechanical properties of TiB_2_ particle reinforced TiAl composites by plasma arc melting process. Mater Sci Eng, A.

[CR4] Huang SC, Hall EL (1991). The effects of Cr additions to binary TiAl-base alloys. Metall Mater Trans A.

[CR5] Kawabata T, Fukai H, Izumi O (1998). Effect of ternary additions on mechanical properties of TiAl. Acta Mater.

[CR6] Kim YW (1994). Ordered intermetallic alloys, part III: gamma titanium aluminides. JOM.

[CR7] Kumagai T, Nakamura M (1996). Effects of aluminum content and microstructure on tensile properties of TiAl alloys. Scr Mater.

[CR8] Lee TW, Lee CH (1999). Microstructure and mechanical properties of TiB_2_/TiAl composites produced by reactive sintering using a powder extrusion technique. J Mater Sci Lett.

[CR9] Liu Q, Nash P (2011). The effect of Ruthenium addition on the microstructure and mechanical properties of TiAl alloys. Intermetallics.

[CR10] Music D, Schneider JM (2006). Effect of transition metal additives on electronic structure and elastic properties of TiAl and Ti_3_Al. Phys Rev B.

[CR11] Ramaseshan R, Kakitsuji A, Seshadri SK, Nair NG, Mabuchi H, Tsudaa H, Matsui T, Morii K (1999). Microstructure and some properties of TiAl–Ti_2_AlC composites produced by reactive processing. Intermetallics.

[CR12] Shu SL, Qiu F, Lü SJ, Jin SB, Jiang QC (2012). Phase transitions and compression properties of Ti_2_AlC/TiAl composites fabricated by combustion synthesis reaction. Mater Sci Eng, A.

[CR13] Shu SL, Xing B, Qiu F, Jin SB, Jiang QC (2013). Comparative study of the compression properties of TiAl matrix composites reinforced with nano-TiB_2_ and nano-Ti_5_Si_3_ particles. Mater Sci Eng, A.

[CR14] Shu SL, Qiu F, Xing B, Jin SB, Wang JG, Jiang QC (2013). Effects of Mn and strain rate on the compression behavior of TiAl alloy fabricated by combustion synthesis and hot press consolidation. Intermetallics.

[CR15] Shu SL, Qiu F, Tong CZ, Shan XN, Jiang QC (2014). Effects of Fe, Co and Ni elements on the ductility of TiAl alloy. J Alloy Compd.

[CR16] Tsujimoto T, Hashimoto K, Nobuki M (1992). Alloy design for improvement of ductility and workability of alloys based on intermetallic compound TiAl. Mater T JIM.

[CR17] VanMeter ML, Kampe SL, Christodoulou L (1996). Mechanical properties of near-titanium aluminides reinforced with high volume percentages of TiB_2_. Scr Mater.

[CR18] Wu XH (2006). Review of alloy and process development of TiAl alloys. Intermetallics.

[CR19] Yang F, Kong FT, Chen YY, Xiao SL (2010). Effect of spark plasma sintering temperature on the microstructure and mechanical properties of a Ti_2_AlC/TiAl composite. J Alloy Compd.

[CR20] Yeh CL, Li RF (2008). Formation of TiAl–Ti_5_Si_3_ and TiAl–Al_2_O_3_ in situ composites by combustion synthesis. Intermetallics.

